# P05.03. How acupuncturists and physicians view the presence of in-patient acupuncture care at Beth Israel Medical Center – a phenomenological study

**DOI:** 10.1186/1472-6882-12-S1-P363

**Published:** 2012-06-12

**Authors:** B Kielczynska, B Kligler

**Affiliations:** 1 Beth Israel Medical Center, Dept of Integrative Medicine, New York City, USA

## Purpose

To better understand the “living experience” of acupuncturists while they provide acupuncture in-patient care and interact with medical staff in a hospital-based Acupuncture Fellowship Program (AFP). Little is known about how acupuncturists and medical staff negotiate cooperation and how the limits of a hospital setting affect efficacy of acupuncture care. Despite the mounting evidence of acupuncture’s effectiveness, its integration into the in-patient setting is limited; we hope that describing challenges that acupuncture integration faces may help move this process forward.

## Methods

We conducted 30-120 minute interviews with five acupuncturists, two physicians and a nurse at Beth Israel Medical Center in NYC, all actively involved in AFP. Our research focused on the question: “How is it for you as an acupuncturist to work in a hospital setting?” We asked participants to describe their experiences, thoughts, and feelings while treating hospital patients and interacting with clinical staff and we analyzed emerging themes with Colaizzi’s phenomenological method. Phenomenology allows researchers to access the meaning of participants’ experience (interpretive paradigm), rather than trying to predict their behavior (empirico-analytical paradigm).

## Results

The following major themes were identified: acupuncture can provide efficacious in-patient care valued by patients; physicians’ support of acupuncture depends more on clinical results than on their understanding of the philosophy behind acupuncture; physicians who receive acupuncture are more likely to advocate for it; different departments of the hospital represent distinct “cultures,” some of which are much more receptive to acupuncture than others.

## Conclusion

The phenomenon of acupuncturists’ experience as they negotiate integration of the traditional East-Asian medicine with modern biomedicine at the BIMC may (1) enrich the roadmap to how acupuncture and other non-biomedical healing traditions can be incorporated into our healthcare system; and (2) provide an example of how current acupuncture and integrative medicine research may be enriched by qualitative methodologies.

